# Evaluating the Effect of Different Intra-Orifice Barriers and Various Bleaching Agents on the Fracture Resistance of Teeth After the Walking Bleach Procedure: An In Vitro Study

**DOI:** 10.7759/cureus.40509

**Published:** 2023-06-16

**Authors:** Geo T D, Saurabh Gupta, Kuldeep Singh Rana, Anisha Kulkarni, Dimple Jadhaw, Neelam Vijaywargiya, Shraddha Pawar, Nilima Pagare

**Affiliations:** 1 Conservative Dentistry and Endodontics, Government College of Dentistry, Indore, IND

**Keywords:** white mineral trioxide aggregate, bulk-fill composite, biodentine, external cervical resorption, intra orifice barriers, intra coronal bleaching

## Abstract

Objective

This study aimed to evaluate the effect of three different commercially available intra-orifice barriers and bleaching agents on root canal-treated teeth.

Materials and methods

Forty-five freshly extracted single-rooted incisors, canine, and premolars were collected and stored in 10% formalin. Root canal procedures were performed on the extracted teeth and these were classified into three groups and three subgroups (n=5). Group 1: resin-modified glass ionomer cement (RMGIC); placed at the level of cemento-enamel junction (CEJ) and cured for 20 seconds. Group 2: Biodentin^TM^ (Septodont Ltd., Saint Maur des Fausse´s, France); powder and liquid were mixed according to the manufacturer's instructions and placed at the level of CEJ, and waited for 15 minutes to set. Group 3: bulk-fill composite; placed at the level of CEJ. Group A was treated with 35% carbamide peroxide (Ultradent Opalescence 35% PF regular). Group B was bleached with 35% hydrogen peroxide (Pola Office). Group C, which was the control group, was treated with distilled water. The bleaching procedure was repeated once every seven days for a period of three weeks. After bleaching, every sample was sectioned 2 mm above the level of CEJ to remove the crown._ _Auniversal testing machine (UTM) was used for the evaluation of the fracture resistance of teeth. Data were analyzed for significance by using analysis of variance (ANOVA) and further pair-wise comparison was performed by pos-hoc analysis. The level of significance was set at p<0.05

Results

There was a significant difference between the fracture resistance of the three materials when bleached using distilled water (p<0.05). The fracture resistance of Group 3 was significantly greater than that of Group 2 and Group 1 (p<0.05). The difference in the fracture resistance between Group 1 and Group 2 was nonsignificant (p>0.05).

Conclusion

Walking bleach performed via bleaching agents 35% carbamide peroxide and 35% hydrogen peroxide leads to a reduction in the fracture resistance of endodontically treated teeth; 35% hydrogen peroxide causes more fracture resistance reduction than carbamide peroxide of the same concentration. The presence of intra-orifice barriers leads to greater fracture resistance and reinforcement of endodontically treated teeth that undergo the walking bleach procedure. Bulk-fill composite can be used as an intra-orifice barrier with good fracture resistance.

## Introduction

Esthetics have become very important in modern society and facial esthetics even more so. A lot of patients, concerned with yellowish or discolored teeth, demand to have whiter and brighter shades for their teeth. Apart from various other reasons for tooth discoloration, traumatic tooth discolorations are also very frequent. Intra-coronal bleaching is the most preferred option for treating these traumatic tooth injuries causing tooth discolorations due to its conservative nature, simplicity, and efficiency. However, this bleaching procedure can lead to some adverse effects such as cervical resorption and reduction in the hardness of tooth structure [1].

Carbamide peroxide and sodium perborate are some of the bleaching agents commonly used in dentistry. These agents disintegrate to release hydrogen peroxide as an active ingredient. Because of its low molecular weight, hydrogen peroxide can penetrate dentin and, by releasing nascent oxygen, can break dimer bonds of organic and inorganic compounds present in the dentin tubules and convert them into colorless compounds by the chemical reduction of the colored agents [2]. Previous studies have shown that bleaching agents reduce the bond strength of the tooth structure [3-7]. There exists a great controversy regarding the fracture resistance of teeth after bleaching. Hence, it is necessary to evaluate as to which bleaching agent has the least adverse effects on tooth hardness.

A protective base is always used to seal the root canal, which prevents the movement of the agents used for bleaching into the pulp canal [2]. Many materials have been proposed as protective bases, including zinc oxide eugenol, zinc phosphate, etc. These days, resin-modified glass ionomer cement (RMGIC)/GIC is the most commonly used intra-orifice sealer. The major drawback of these materials is the risk of microleakage and subsequent external cervical resorption associated with them [1]. Hence, most of the studies focus on measuring the microleakage of the materials, but the reduction in strength is often overlooked. Therefore, it is necessary to compare the efficacy of newer restorative materials used as intra-orifice barriers and find out which is the most efficacious intra-orifice barrier that also reinforces the tooth.

Bulk-fill composite, mineral trioxide aggregate (MTA), and Biodentin^TM^ (Septodont Ltd., Saint Maur des Fausse´s, France) are some other restorative materials that can be used as an intra-orifice barrier. The full-body bulk-fill composites are regarded as the only true bulk-filling type since the whole restoration can be placed at once without requiring any layering. These materials generally have higher filler loads, which make them highly viscous; for this reason, these materials are often referred to as paste-like bulk-fill composites [8]. Because of this added advantage, bulk-fill composites can be used as an intra-orifice barrier for the walking bleach procedure [9].

MTA has been evaluated for a wide variety of applications [10]. MTA is a powder that consists of fine hydrophilic particles set in the presence of moisture. Superior marginal adaptation and the ability to resist marginal leakage are the important properties of MTA. Additionally, the finer particle sizes make the handling of material easier [11]. However, despite the wide range of potential applications, few studies have been conducted to evaluate MTA as a coronal barrier [12-14]. Biodentin is a tricalcium silicate (Ca_3_SiO_5_)-based inorganic restorative commercial cement and it has been advertised as a "bioactive dentine substitute". This material is claimed to possess better physical and biological properties compared to other tricalcium silicate cement [15]. Biodentine is a powder and liquid system where the powder is composed of tricalcium silicate (main component), calcium carbonate (filler material), zirconium oxide (radiopacifier), dicalcium silicate (traces), calcium oxide (traces), and iron oxide (traces), while the liquid is an aqueous solution of a hydrosoluble polymer (water-reducing agent) with calcium chloride (decreases the setting time) [16]. This bioceramic material is suitable as an intro-orifice barrier because of its properties like remineralization of dentin, mechanical properties similar to those of normal tooth structure, ease of use and handling, short setting time, resistance to leakage, and biocompatibility [17].

Different bleaching agents and intra-orifice barriers exert various kinds of effects on the endodontically treated tooth, especially on fracture resistance. This study evaluates the effect of three commercially available intra-orifice barriers and bleaching agents on root canal-treated teeth. The null hypothesis of the present study was as follows: there is no effect on the fracture resistance of teeth with the use of different bleaching agents and intra-orifice sealers have no impact on the fracture resistance of teeth.

## Materials and methods

**Figure 1:**
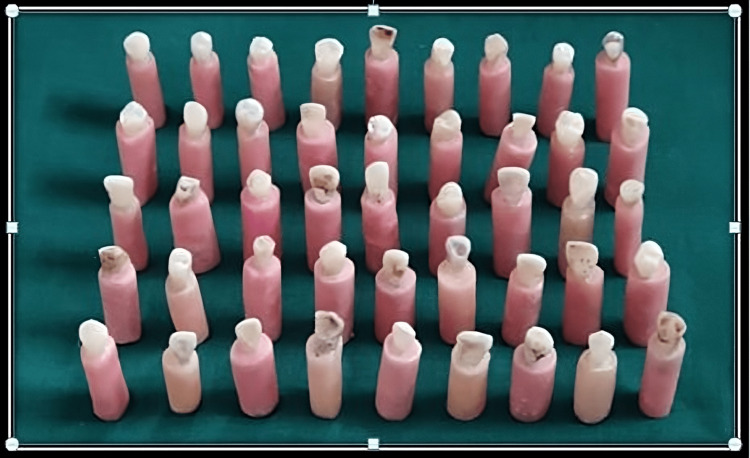
Extracted teeth mounted in acrylic blocks

Forty-five freshly extracted intact human single-rooted incisors, canine, and premolars were disinfected by immersing them in 5.25% sodium hypochlorite solution for 10 minutes and stored in 10% formalin at room temperature. Teeth without developmental anomalies and within the required dimension of 4-8 mm mesiodistally and 4-9 mm labiolingually were included in the study. Those teeth with severely curved and calcified root canals, previously endodontically treated teeth, and teeth with enamel or dentine fracture/caries/damages were excluded from the study. Before root canal treatment and bleaching procedure, teeth were fixed in a cold cure resin cylinder with the labial aspect of the tooth perpendicular to the table surface with a width of 1.5 cm and height of 2.5 cm in such a way that 5 mm of root structure was available outside the resin block (Figure 1).

Preoperative radiographs were taken using radiovisiography (RVG). Access cavity on the teeth was prepared using endo-access bur, and canal negotiation was done with number 10 K-file followed by radiographic confirmation of the working length. Biomechanical preparation was performed with rotary instrumentation. Apical preparation for all teeth was done up to F1, with ProTaper Gold (diameter: 0.20 mm; taper: 7%). Sodium hypochlorite 5.25% and 17% ethylene diamine tetraacetic acid (EDTA) were used as root canal irrigants for the entire procedure. After cleaning and shaping, radiographs were taken with the master cone in the root canal. Obturation was done by lateral compaction using gutta-percha, with zinc oxide eugenol as a sealer. After radiographic evaluation of obturated teeth, 3 mm of coronal gutta-percha was removed from the cemento-enamel junction (CEJ) using a peeso reamer. Samples were again evaluated radiographically.

Then, the endodontically treated teeth were randomly categorized into three groups. In each group, different intra-orifice barriers were placed at the level of CEJ for a thickness of 3 mm. Group 1: resin-modified glass ionomer cement (RMGIC); placed at the level of CEJ and cured for 20 seconds. Group 2: Biodentine; powder and liquid were mixed according to the manufacturer's instructions and placed at the level of CEJ and waited for 15 minutes to set. Group 3: bulk-fill composite; placed at the level of CEJ and light-cured for 20 seconds. Radiographs were taken to confirm the thickness of the intra-orifice barrier. After that, each group's samples were randomly divided into three subgroups: A, B, and C (n=5). Group A was treated with 35% carbamide peroxide (Ultradent Opalescence 35% PF regular). Group B was bleached with 35% hydrogen peroxide (Pola Office). Group C, which was the control group, was treated with distilled water. In each group, bleaching material was placed inside the pulp chamber and access was restored with temporary restoration (Cavit G). The bleaching procedure was repeated once every seven days for a period of three weeks. During this period, teeth were incubated in normal saline at 37 °C. After bleaching, every sample was sectioned 2 mm above the level of CEJ by using a diamond disc to remove the crown.

Measurement of fracture resistance

**Figure 2:**
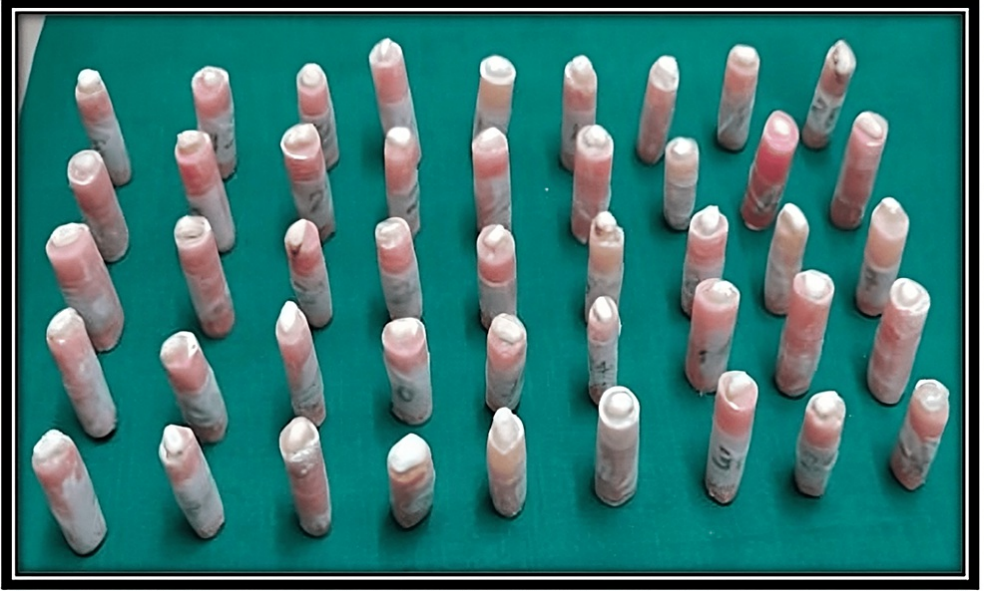
Decoronated samples after bleaching

**Figure 3:**
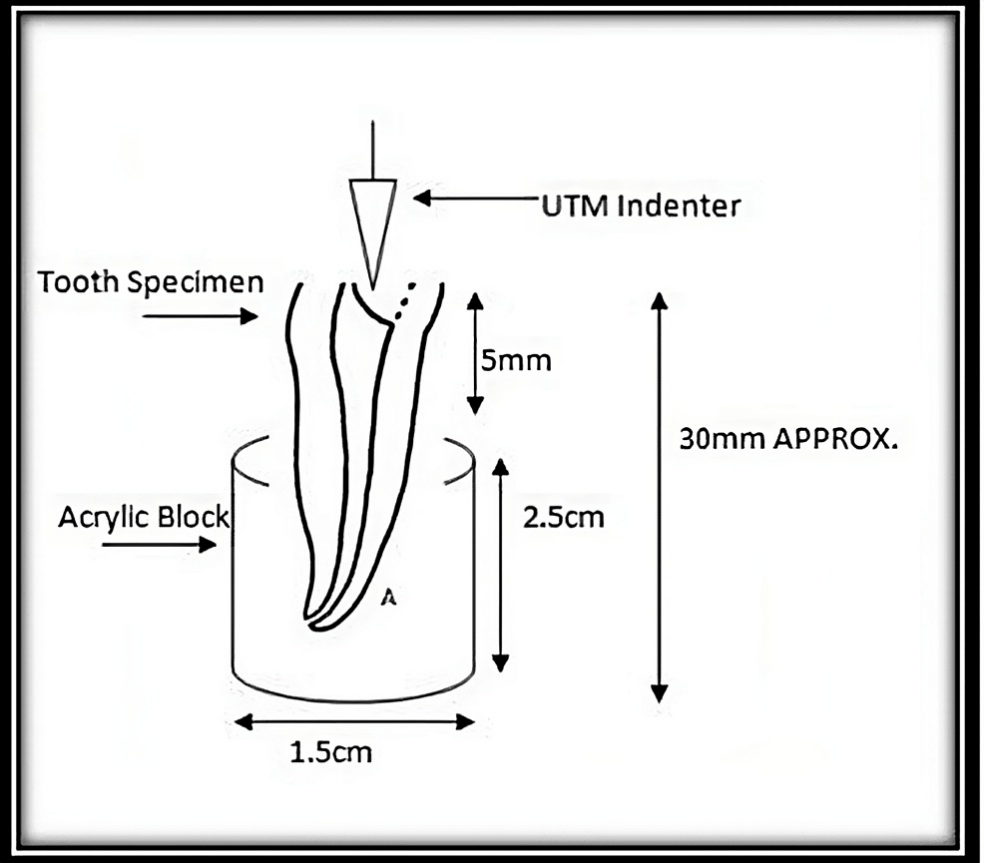
Graphical representation of the measurement of fracture resistance by using a universal testing machine (UTM)

**Figure 4:**
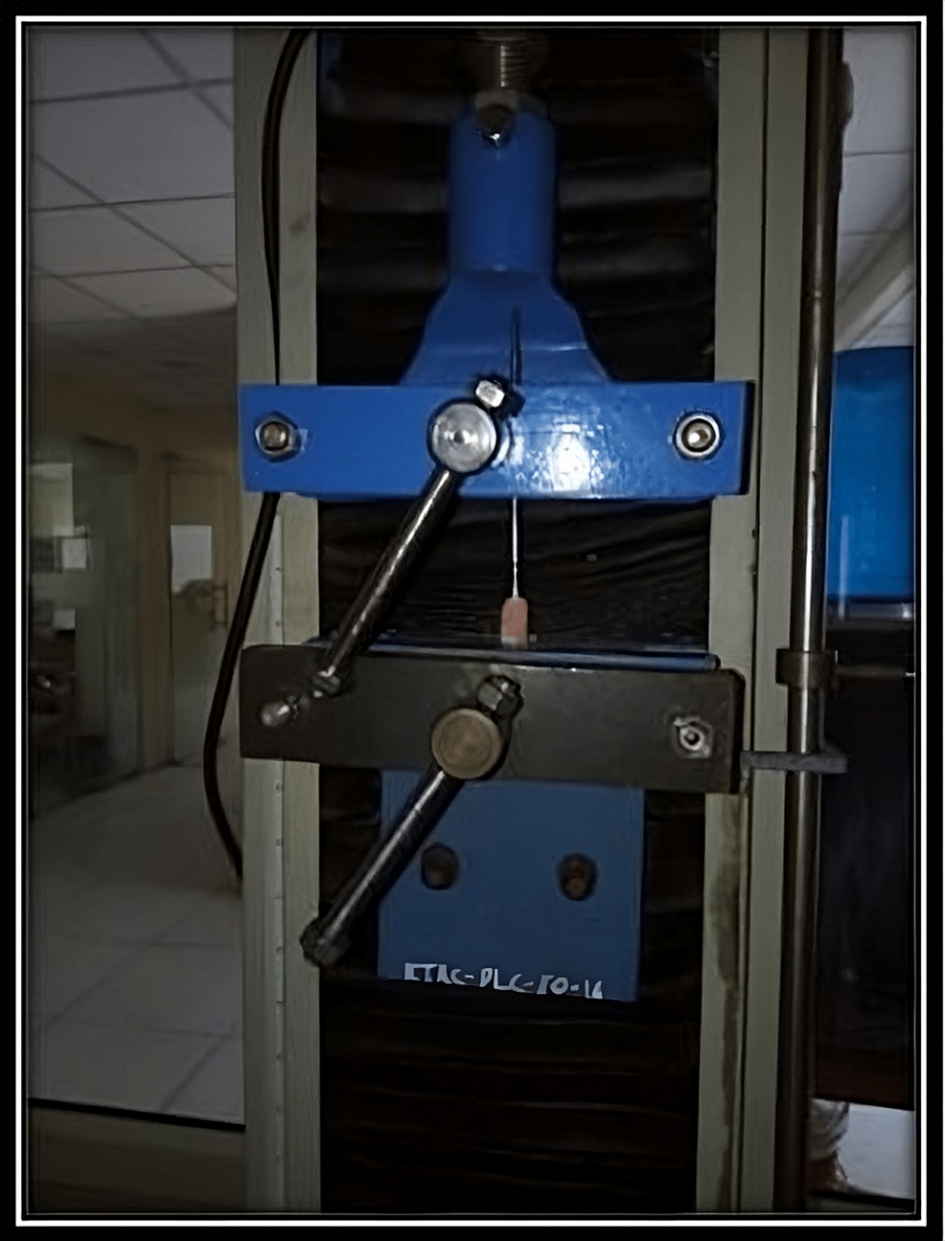
Measuring fracture resistance of samples by using a universal testing machine (UTM)

A universal testing machine (UTM) was used for the evaluation of fracture resistance of teeth. The perpendicular force was applied to the center of the decoronated tooth at a rate of 1 mm/minute by using a blunt indenter (Figures 2-4).

Statistical analysis

Data were entered into an Excel sheet and analyzed using IBM SPSS Statistics version 21.0 (IBM Corp., Armonk, NY). Data were analyzed for probability distribution using the Kolmogorov-Smirnov test and were found to be normally distributed. Thus, the parametric test was applied. Descriptive statistics were performed. Data were presented as mean and standard deviation (SD). The comparison of the fracture resistance was performed using one-way analysis of variance (ANOVA). A p-value <0.05 was considered statistically significant.

## Results

In Group 1, the mean fracture resistance of RMGIC samples without bleaching (395.8 ±51.59651) was the highest followed by those with bleaching using hydrogen peroxide (318.1 ±79.84545) and those with bleaching using carbamide peroxide (300.6 ±132.89394) (Table 1). The difference in the fracture resistance among the samples belonging to the three subgroups of Group 1 was statistically nonsignificant (p>0.05). In Group 2, the average fracture resistance of Biodentine samples with bleaching using carbamide peroxide (325.4 ±126.94190) was the highest followed by those without bleaching (322.5 ±112.60504) and those with bleaching using hydrogen peroxide (316.3 ±99.76983) (Table 1). The difference in the fracture resistance among the samples belonging to the three subgroups of Group 2 was statistically nonsignificant (p>0.05). In Group 3, the mean fracture resistance of bulk-filled samples without bleaching (376.0 ±48.97959) was the highest followed by those with bleaching using carbamide peroxide (346.4 ±42.93367) and those with bleaching using hydrogen peroxide (613.6 ±53.83997). The difference in the fracture resistance among the samples belonging to the three subgroups of Group 3 was statistically significant (p<0.05). The fracture resistance of non-bleached bulk-filled samples was significantly greater than those that had been bleached using carbamide peroxide and hydrogen peroxide (p<0.05). The fracture resistance between non-bleached bulk-filled samples and those that had been bleached using carbamide peroxide and those bleached using hydrogen peroxide was not found to differ significantly (p>0.05) (Table 2).

**Table 1:** Comparison of fracture resistance ^ª^One-way ANOVA; *p-value <0.05 was considered statistically significant

	Mean	Standard deviation	F value	P-valueª	
Group 1A	300.6000	132.89394	1.443	0.274	
Group 1B	318.1000	79.84545			
Group 1C	395.8000	51.59651			
Total	338.1667	97.27385			
Group 2A	325.4080	126.94190	0.008		0.992
Group 2B	316.3040	99.76983			
Group 2C	322.5420	112.60504			
Total	321.4180	105.29188			
Group 3A	376.0000	48.97959	45.073		0.001*
Group 3B	346.4000	42.93367			
Group 3C	613.6220	53.83997			
Total	445.3407	131.78557			

**Table 2:** Comparison of fracture resistance between different barriers with various bleaching agents ^ª^One-way ANOVA; *p-value <0.05 was considered statistically significant

	Mean	Standard deviation	F value	P-valueª
Group 1A	300.6000	132.89394	0.612	0.558
Group 2A	325.4080	126.94190		
Group 3A	376.0000	48.97959		
Group 1B	318.1000	79.84545	0.235	0.794
Group 2B	316.3040	99.76983		
Group 3B	346.4000	42.93367		
Group 1C	395.8000	51.59651	18.851	0.001*
Group 2C	322.5420	112.60504		
Group 3C	613.6220	53.83997		

Among the samples bleached using carbamide peroxide, the fracture resistance of bulk-filled (376.0 ±132.89394) was the highest followed by Biodentine (325.4 ±126.94190) and RMGIC (300.6 ±132.89394) (Table 2). There was no significant difference in the fracture resistance among the three materials when bleached using carbamide peroxide (p>0.05). Regarding samples bleached using hydrogen peroxide, the fracture resistance of bulk-filled (346.4 ±42.93367) was the highest followed by RMGIC (318.1 ±79.84545) and Biodentine (316.3 ±99.76983). There was no significant difference in fracture resistance among the three materials when bleached using hydrogen peroxide (p>0.05). Among the samples with no bleaching, the fracture resistance of bulk-filled (613.6 ±53.83997) was the highest followed by RMGIC (395.8 ±112.60504) and Biodentine (322.5 ±112.60504) (Table 2). There was a significant difference in fracture resistance among the three materials when bleached using distilled water (p<0.05).

**Figure 5:**
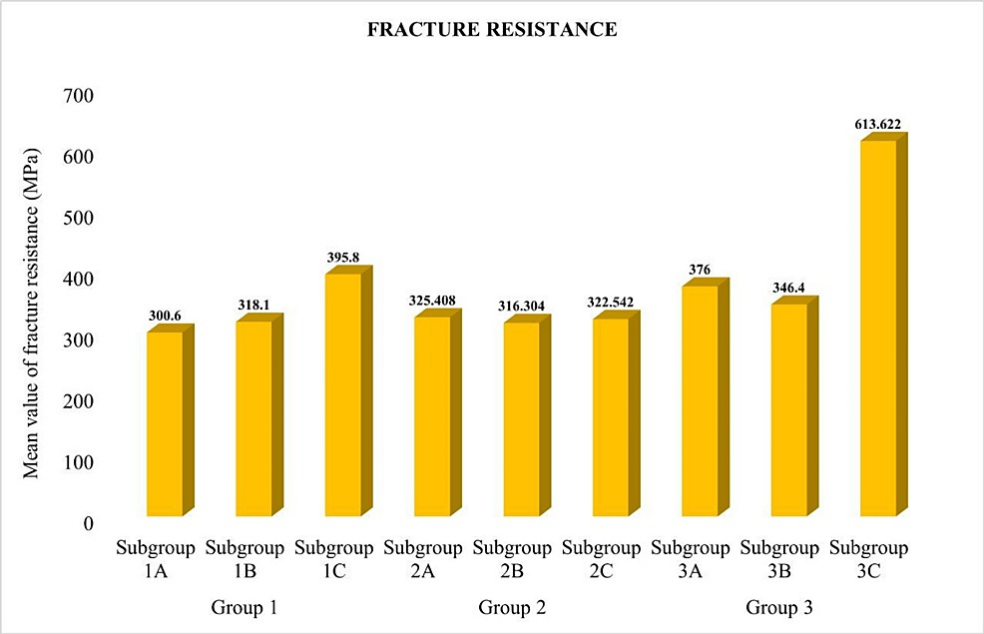
Comparison of fracture resistance among the three groups

The fracture resistance of bulk-filled was significantly greater than that of Biodentine and RMGIC (p<0.05). However, the fracture resistance between RMGIC and Biodentine was found to differ nonsignificantly (p>0.05) (Figure 5).

## Discussion

This in vitro study evaluated the effect of various bleaching agents and intra-orifice barriers on the fracture resistance of endodontically treated teeth. Walking bleach was performed with either 35% carbamide peroxide or 35% hydrogen peroxide for a period of three weeks. Root canal orifices were sealed at the level of CEJ with three different barriers: RMGIC, Biodentine, and bulk-fill composite; 35% carbamide peroxide or hydrogen peroxide did not have any statistically significant effect on fracture resistance of teeth. Except for bulk-fill composite, the intra-orifice barriers did not significantly affect the fracture resistance. When the bulk-fill composite was used as an orifice barrier, the fracture resistance of teeth was higher than that with RMGIC and Biodentine. Carbamide peroxide or hydrogen peroxide with bulk-fill composite did not show a statistically significant difference in the reduction of fracture resistance. The first null hypothesis of the study, i.e., there is no effect on fracture resistance of teeth with the use of different bleaching agents, has not been rejected; however, the second hypothesis, i.e., intra-orifice sealers have no impact on fracture resistance of teeth, has been rejected.

In the walking bleach treatment, either by carbamide peroxide or hydrogen peroxide, there was a mean decrease in the fracture resistance of endodontically treated teeth. In the present study, the bleaching procedure was performed three times with an interval of seven days. Walking bleach procedure cause more reduction in fracture resistance of teeth due to the residues of bleaching agents that remains within the dentinal tubules, and continuous mechanical and thermal alterations that can occur intra-orally. Our study results are in line with those of Leonardo Rde et al. [18]. They evaluated the fracture resistance of teeth subjected to several internal bleaching protocols using 35% hydrogen peroxide, 37% carbamide peroxide, and 15% hydrogen peroxide with titanium dioxide nanoparticles photoactivated by LED-laser or sodium perborate. After four sessions of bleaching, with seven-day intervals, there was a reduction in fracture resistance of endodontically treated teeth, but there were no differences between different procedures.

The current study used single-rooted mature maxillary and mandibular incisors, canine, and premolars. To avoid bias, the teeth were equally distributed by number and type in each group. In all samples, the root canal procedure was performed by using ProTaper Gold files, and the canal debridement was done with 5.25% sodium hypochlorite and 17% EDTA. Root canal apical preparation was completed with ProTaper Gold F1 (0.20-mm tip diameter and 7% taper).

Most of the walking bleach procedures will give satisfactory results within three to four visits with an intermittent changing of the bleaching agent in three to seven days between each visit [19]. In the present study, bleaching was done three times with an interval of seven days between each session. During the bleaching period, the samples were incubated at 37 °C, the normal human body temperature, in a normal saline solution; 35% hydrogen peroxide is an effective bleaching agent for the nonvital tooth bleaching procedure [20]. Nevertheless, high concentrations should be used with caution, in order to avoid increasing risk of root resorption [21]. Carbamide peroxide is an organic compound containing hydrogen peroxide and urea. In vitro study conducted by Tam et al. showed that carbamide peroxide has a bleaching ability similar to that of hydrogen peroxide [22]. During bleaching, the long-chain organic molecules are transformed into carbon and water and are released together with nascent oxygen [23]. Hydroxyl ions liberated from hydrogen peroxide carry out the bleaching action, while 10% carbamide peroxide breaks down into 3.35% hydrogen peroxide and 6.65% urea. The urea further breaks down into ammonia and water, which may provide some beneficial side effects because it tends to increase the pH of the solution [24].

Root resorption and reduction in fracture resistance are the disadvantages associated with nonvital tooth bleaching [25]. In the current study, 35% carbamide peroxide showed more reduction in the mean fracture resistance of teeth than 35% hydrogen peroxide. Another in vitro study by Tam et al. on the effect of different concentrations of carbamide peroxide and hydrogen peroxide on fracture resistance of dentine also concluded that carbamide peroxide bleaching showed more reduction in fracture resistance than hydrogen peroxide [26]. Percolation of the bleaching agent through the interface between intra-orifice barrier-root dentin wall and subsequent penetration of oxygen free radicals to the dentinal tubules affects not only the inorganic components of the dental hard tissues through acidic demineralization but also attacks the relatively rich organic substance of the dentin. This effect on the organic substance might be due to collagen denaturation [27].

Microleakage of the bleaching agent is the primary reason for complications like cervical resorption and reduction in fracture resistance [28]. Intra-orifice barriers act as a separator between the bleaching agent-filled pulp chamber and obturated root canal. The selection of orifice sealers should be based on their properties: retention, resistance, and microleakage of the restorative material [29]. With recent advancements in the field of adhesive dentistry, newer restorative materials offer a higher percentage of bonding and increased retention [30]. The intra-orifice barriers affect the fracture strength of the tooth by replacing the dentin lost by the endodontic procedure [29]. RMGIC, Biodentine, and bulk-fill composite were the three orifice sealers used in this in vitro study. The era of RMGIC began in the late 1980s; it contains some methacrylate components common in resin composites. It offers an acceptable coronal seal of more than 90 days, as reported by Tselnik et al., due to the superior performance of RMGIC thanks to water sorption by the material, resulting in setting expansion and, consequently, a better seal is achieved [13]. It does not require any pre-conditioning of dentin and can adhere to it. Another useful property of RMGIC is the release of fluoride [31]. RMGIC has high flexural strength and modulus of elasticity, and modulus of elasticity values that are similar to dentin; the material can withstand large amounts of stress before transmitting the load to the root [32]. In the current study, RMGIC did not show any significant difference in fracture resistance when compared with Biodentine, but it was significantly lower when compared to bulk-fill composites.

Biodentine could be used as an intra-orifice restorative material due to its properties of remineralization of dentin, mechanical properties similar to those of dentin, ease of use and handling, short setting time, resistance against leakage, and its nontoxic nature. In this study, the combination of Biodentine and carbamide peroxide showed higher fracture resistance than that with hydrogen peroxide. However, when compared with bulk-fill composite, it showed less fracture resistance.

Tetric-N-Ceram, a bulk-fill composite, is a nanohybrid composite recently introduced in restorative dentistry. The mouldable composite resin can be placed in increments of up to 4 millimeters using the bulk-filling technique. The light activator Ivocerin is responsible for ensuring the complete cure of the restoration. Compared with conventional light initiators, the Ivocerin polymerization booster is much more reactive. Therefore, polymerization is initiated even in very deep cavities and the material is fully cured. Short polymerization time and long working time are the two important properties of Tetric-N-Ceram. Tetric N-Ceram bulk-fill cures quickly in only 10 seconds (>1,000 mW/cm^2^). Due to its smooth consistency, Tetric-N-Ceram bulk-fill readily adapts to cavity walls. A specially conditioned composite filler, also called a shrinkage stress reliever, keeps shrinkage and shrinkage stress during polymerization to a minimum. It acts like a spring to dampen the forces generated during shrinkage [33]. The presence of modified high-molecular-weight urethane dimethacrylate (UDMA) also contributes to less shrinkage and hence shrinkage stress. Among the three intra-orifice barriers, bulk-fill composite showed a greater amount of fracture resistance than RMGIC and Biodentine. This could be attributed to the added advantages of Tetric-N-Ceram. Bulk-fill composite showed statistically nonsignificant (p>0.05) greater fracture resistance when compared with carbamide peroxide than with hydrogen peroxide.

This in vitro study tried to use the most advanced restorative material for sealing root canal orifices and for comparing the fracture resistance of teeth. Additionally, the study used commonly utilized bleaching agents, i.e., carbamide peroxide and hydrogen peroxide, which are present in most of the commercially available bleaching systems. Dimensions of the samples were standardized prior to the study. Standardized endodontic procedures performed similarly for all the samples and simulated the walking bleach procedure in terms of duration were the strengths of the study. However, small sample sizes and the inability to simulate the intra-oral conditions were the main limitations of the study.

MTA is an alternative option for intra-orifice barriers [10,34]. In most studies, MTA has shown satisfactory microleakage protection than other restorative materials [35]; however, it is associated with a long setting time [36] and poor handling [37]. The reinforcement to the endodontically treated teeth provided by MTA is also questionable. In the in vitro study conducted by Aktemur Türker et al. on immature necrotic teeth with different intra-orifice sealers, MTA showed the lowest fracture strength than other materials: composite resin and RMGIC [38]. The study by Özyurek et al., which analyzed the push-out bond strength of intra-orifice sealers, concluded that ProRoot MTA and Biodentine calcium silicate cement have lower bond strength than SureFil SDR and EverX Posterior bulk-fill composite resins [39].

Future studies should focus on the microleakage of bleaching agents that can occur through different orifice sealers and the level of percolation that can happen.

## Conclusions

Within the limitations of this in vitro study, the following conclusions can be drawn: walking bleaching performed via bleaching agents 35% carbamide peroxide and 35% hydrogen peroxide leads to a reduction in the fracture resistance of endodontically treated teeth; 35% hydrogen peroxide causes more fracture resistance reduction than carbamide peroxide of the same concentration but the difference is nonsignificant. The presence of intra-orifice barriers leads to greater fracture resistance and reinforcement of endodontically treated teeth that undergo the walking bleach procedure. Tetric-N-Ceram bulk-fill composite can be used as an intra-orifice barrier with good fracture resistance.

Microleakage caused by the intra-orifice barriers should be evaluated in future studies. Also, further research is needed to devise a more biocompatible restorative material that provides greater retention, resistance, and the least microleakage to be used as an intra-orifice barrier.
